# Membrane Vesicles of *Enterococcus faecalis*: In Vitro Composition Analysis and Macrophage Inflammatory Response Under Different pH Conditions

**DOI:** 10.3390/microorganisms13061344

**Published:** 2025-06-10

**Authors:** Zijian Yuan, Wenling Huang, Poukei Chan, Jiani Zhou, Jingheng Liang, Lihong Guo

**Affiliations:** Hospital of Stomatology, Guanghua School of Stomatology, Sun Yat-sen University, Guangzhou 510055, China; yuanzj5@mail2.sysu.edu.cn (Z.Y.); huangwling3@mail2.sysu.edu.cn (W.H.); chanpk@mail2.sysu.edu.cn (P.C.); zhoujn7@mail2.sysu.edu.cn (J.Z.); liangjh79@mail2.sysu.edu.cn (J.L.)

**Keywords:** *Enterococcus faecalis*, membrane vesicles, proteomics, metabolomics, macrophages, immuno-inflammatory responses

## Abstract

*Enterococcus faecalis* (*E. faecalis*) is one of the most detected bacteria in persistent apical periodontitis (PAP), with alkaline tolerance enabling post-treatment survival. In this study, we will investigate how alkaline conditions alter proteomic and metabolomic profiles of *E. faecalis* membrane vesicles (MVs) and preliminarily investigate the role of MVs of *E. faecalis* in the regulation of macrophage inflammatory response. *E. faecalis* MVs were characterized using transmission electron microscopy and nanoparticle tracking analysis under varying pH conditions. MVs’ proteomic and metabolomic profiling across pH levels was compared. The effects of *E. faecalis* MVs on human dTHP-1 macrophages were evaluated using CCK-8 metabolic activity assays and ELISA-based quantitative analysis of inflammatory cytokines. In this study, the presence of *E. faecalis* MVs was verified, and the alkaline environment of pH 9.0 did not alter their production. Through proteomic and metabolomic analysis, we observed that ATP synthase and stress proteins, as well as lysine degradation and tryptophan metabolism pathways, were significantly enriched in the MVs at pH 9.0. Finally, we observed that both *E. faecalis* MVs at pH 7.0 and pH 9.0 could dose-dependently inhibit the activity of dTHP-1 cells. *E. faecalis* MVs promote the secretion of IL-6, TNF-α, IL-1β, IL-1ra, and TGF-β by macrophages. Compared to pH 7.0, pH 9.0 *E. faecalis* MVs have a reduced effect on IL-1ra and TGF-β secretion. Additionally, we observed a significant increase in the IL-1β/IL-1ra ratio after treatment with *E. faecalis* MVs. Our study indicated that *E. faecalis* can produce MVs in pH 7.0 and pH 9.0 environments. ATP synthase, stress proteins, as well as lysine degradation and tryptophan metabolism pathways, were significantly enriched in pH 9.0 MVs. Furthermore, *E. faecalis* MVs could promote inflammatory responses in macrophages and dose-dependently inhibit the viability of dTHP-1 cells.

## 1. Introduction

Apical periodontitis is a condition of inflammation in the periapical region resulting from an infection of the dental pulp within the root canal system [1]. Irreversible infection and necrosis of the pulp can lead to bone loss in the apical region if the root canal is not effectively treated [2]. Primary apical periodontitis occurs when necrotic pulp tissues become colonized by microbes. Persistent infection of improperly treated root canals will lead to secondary apical periodontitis (SAP) [3]. In addition, the chronic inflammation of periapical tissue and failure of bone healing after repeated routine RCT may rise up to the development of apical periodontitis, ultimately resulting in tooth loss, posing a significant clinical challenge [4].

Residual microorganisms located in the apical region of the root canal system are the primary factor contributing to post-treatment apical periodontitis [5,6], regardless of whether the treatment was performed inadequately or appropriately. Studies have shown that *E. faecalis* is one of the most frequently isolated species in failed root canal-treated teeth [7]. And there is a higher rate of retreatment failure when *E. faecalis* is present in the root canals during root filling [8].

RCT involves a series of technical procedures, including precise shaping and cleaning of the infected root canal, irrigation and disinfection, root canal filling with biocompatible materials, and restoration of tooth defects after RCT [9]. Sodium hypochlorite (NaClO) and ethylene diamine tetraacetic acid (EDTA) are frequently used as root canal rinsing solutions [10], whereas calcium hydroxide (Ca(OH)_2_) is commonly utilized as a disinfectant agent [11].

*E. faecalis* maintains its ability to infect root canals even after undergoing complete RCT due to its colonization within the root canal and tolerance under alkaline conditions [12]. Several studies have indicated that gelatinase (Gel), serine protease (Spr), and collagen-binding protein (Ace) enhance the capability of *E. faecalis* to adhere to the walls of root canals [13]. Other studies have indicated that *E. faecalis* has the ability to enter dentinal tubules [8,14,15]. Sigusch et al. developed a standardized dentin model by using artificial SiO/SiO_2_ microtubes and found that *E. faecalis* was able to penetrate and reproduce within the microtubes in a short time [16]. Therefore, although mechanical and chemical preparation of the root canal significantly reduces bacterial infection [17], eliminating *E. faecalis* from dentinal tubules remains a challenging task [18]. Dressings with Ca(OH)_2_ were reported to show only minor effects on completely suppressing *E. faecalis* inside the dentinal tubules [19]. The remarkable resistance displayed by *E. faecalis* towards alkaline environments may be attributed to its various abilities, such as increased cell surface hydrophobicity, Na^+^-K^+^ ATPase activity, up-regulation of stress response genes, and formation of biofilms [20,21]. However, further research is required to establish the potential contribution of other variables.

MVs are diverse membrane formations originating from cellular sources [22]. Gram-positive (G^+^) bacteria are surrounded by a thick and rigid cell wall that was previously believed to prevent the release of MVs, leading to the neglect of G^+^ bacteria MVs. In 2009, the release of bilayer MVs from *Staphylococcus aureus* (*S. aureus*) was confirmed through electron microscopy [23], thereby demonstrating that G^+^ bacteria can also release MVs. Subsequent studies have also shown that MVs are released from a variety of G^+^ bacteria, including *Bacillus subtilis* (*B. subtilis*), *Listeria monocytogenes* (*L. monocytogenes*), *Clostridium perfringens* (*C. perfringens*), and *Enterococcus* spp. [24,25,26,27,28,29,30,31]. Numerous studies have demonstrated the significant roles of G^+^ bacterial MVs in virulence traits, cell communication, response to stress environment and host immunomodulation [23,32,33,34]. Thus, bacterial MVs have attracted considerable attention for their potential in antibacterial therapy. However, the correlation between MVs and alkaline tolerance of *E. faecalis* are currently unexplored areas yet.

In this study, we purified MVs released from *E. faecalis* in neutral and alkaline environments and analyzed the changes in the proteomic profiles and metabolites of *E. faecalis* MVs in alkaline environments. Meanwhile, the effects of MVs produced by *E. faecalis* under neutral and alkaline environments on the activity and immune-inflammatory responses of macrophages were compared. This will provide a basis for elucidating the pathogenic mechanism of persistent *E. faecalis* infection in apical periodontitis and provide insights and targets for the prevention and treatment of apical periodontitis.

## 2. Material and Methods

The manuscript of this laboratory study has been written according to the Preferred Reporting Items for Laboratory Studies in Endodontology (PRILE) 2021 guidelines [35].

### 2.1. Bacterial Strain and Growth Condition

*E. faecalis* (OG1RF ATCC 47077) was obtained from the Guangdong Detection Center of Microbiology (GDDCM) and was utilized in the experiments and preserved at −80 °C in a solution consisting of 50% glycerol. The bacteria were cultured in tryptic soy broth (TSB) with the following composition: 1.7% tryptone, 0.3% polypeptone, 0.1% yeast extract (Huankai Biology, Guangzhou, China), 0.5% NaCl, 0.25% glucose, and 0.25% dipotassium phosphate, and then incubated aerobically at 37 °C for a period of 10 h. The media with varying pH levels (pH 7.0, pH 9.0, and pH 10.0) were adjusted using NaOH and KH_2_PO_4_ and sterilized through a filter unit with a pore size of 0.22 μm (Millipore, Millex-GP, Burlington, MA, USA). The pH of the bacterial solution was monitored at 1 h intervals, with readings taken at a wavelength of 600 nm using an enzyme-linked immunosorbent assay reader (Tecan, Männedorf, Switzerland) to plot the growth curve of *E. faecalis*. Weigh 6 g of TSB powder and 3 g of agar powder with an electronic balance, dissolve them in 200 mL of pure water with stirring at low temperature, autoclave at 121 °C, and cool them to 65 °C in a water bath to prepare TSB plates. After *E. faecalis* has grown to the end of the exponential phase, 1 mL of *E. faecalis* bacterial suspensions with pH 7.0 and pH 9.0 is respectively taken. After gradient dilution, the dilutions are spotted onto TSB agar plates. The colonies of *E. faecalis* can be observed to grow out after an incubation period of 8 to 10 h. Then, the colony-forming units per milliliter (CFU/mL) of the two groups are counted.

### 2.2. Preparation of MVs-Enriched Fraction

MVs enrichment from *E. faecalis* culture supernatants was performed using sequential centrifugation. Firstly, *E. faecalis* after a 9 h incubation was centrifuged at 10,000× *g* for 30 min at 4 °C, then the supernatant was collected and sterilized by a 0.22 μm sterile filter (Millipore, Millex-GP, Burlington, MA, USA). Sterile supernatant was added to an ultrafiltration tube (100 kDa) and centrifuged at 4000× *g* for 30 min at 4 °C. The upper chamber concentrate was collected and added to an ultracentrifuge tube (Beckman, Brea, CA, USA), which was turned in a Ty90 Ti ultracentrifuge (Beckman, Optima XE-100, Brea, CA, USA) and centrifuged at 100,000× *g* for 70 min at 4 °C to obtain an MVs precipitate. The MVs-enriched fraction was washed with PBS and collected again by ultracentrifugation.

The MVs precipitate was resuspended in OptiPrep buffer to obtain 2 mL of 40% OptiPrep solution, then added to the bottom of an ultracentrifuge tube (Beckman, 13.2 mL, Brea, CA, USA). Hierarchically, 2 mL of 35% OptiPrep solution, 2 mL of 30% OptiPrep solution, 2 mL of 25% OptiPrep solution, and 2 mL of 20% OptiPrep solution were successively added. After centrifugation at 200,000× *g* for 120 min at 4 °C, MVs sample rings between the 25% OptiPrep separation solution layer and the 30% OptiPrep separation solution layer were collected. MVs were resuspended in PBS, then added to ultracentrifuge tubes and centrifuged at 100,000× *g* for 70 min at 4 °C to remove excess OptiPrep solution. MVs precipitate was collected and resuspended using 1 mL PBS. Purified MVs were inoculated onto a TSB agar plate for 24 h to determine the sterility of MVs samples. BCA protein quantification kit (Beyotime, Shanghai, China) was used to detect the protein concentration of MVs samples before transmission electron microscopy and proteomic analysis.

### 2.3. Transmission Electron Microscopy (TEM)

The GDDCM was commissioned to carry out TEM observation of MVs, and the specific procedures were as follows. Five microliters of MVs samples of the neutral environment (pH 7.0) and the alkaline environment (pH 9.0) was dispersed and suspended by an ultrasonic dispensator, dropped on the polymethyl vinyl acetate lipid support membrane placed on a 75-mesh copper mesh, incubated for 5 min, and washed 4 times with pure water. A mixture of 2% methylcellulose and 3% uranyl acetate was prepared with a ratio of 9:1. Samples were negatively stained in an ice bath for 2 min and dried at room temperature. MVs released by *E. faecalis* in different pH environments were observed and photographed using TEM H-7650 (Hitachi, Tokyo, Japan).

### 2.4. Nanoparticle Tracking Analysis (NTA)

Ten microliters of pH 7.0 and pH 9.0 *E. faecalis* MVs samples was placed into an ice bath separately. Used double-steamed water to clean the Nanoparticle Tracking Analyzer Zetaview (Particle Metrix, Ammersee, Germany) until the software detected the interface particle value < 3. Pre-testing diluted sample multiples enabled the software to detect interfacial sample particle values between 50 and 400 particles/frames. After injecting 5 mL of diluted sample solution, the camera scanned 11 positions and captured 30 frames at each position. The video was analyzed using the built-in ZetaView software 8.05.14.SP7. The analysis was conducted using specific parameters, including a maximum area of 1000, a minimum area of 10, a minimum brightness of 30, and a laser wavelength of 488 nm.

### 2.5. Proteomics

*E. faecalis* MVs were resuspended in 100 μL PBS, and 10 μL RIPA lysate (Beyotime, Shanghai, China) was added and lysed for 5 min on ice. The BCA Protein Assay kit (Bio-Rad, Hercules, CA, USA) was utilized to quantify the total protein amount. Protein digestion was carried out using trypsin following the filter-aided sample preparation (FASP) procedure. Two hundred micrograms of MVs protein was mixed with 30 μL of SDT buffer (4% sodium dodecyl sulfate (SDS), 100 mM DTT, 150 mM Tris-HCl pH 8.0), and the detergent, DTT, and other reagents were eliminated through repeated ultrafiltration (10 kDa, Microcon units) using UA buffer (8 M urea, 150 mM Tris-HCl pH 8.0). The low-molecular-weight fractions were eliminated through multiple rounds of ultrafiltration (10 kDa), and the cysteine residues were blocked by adding 100 μL of iodoacetamide and allowing it to incubate in darkness for 30 min. Subsequently, the protein suspension was digested using 4 μg of trypsin (Promega, Fitchburg, WI, USA) in 40 μL of 25 mM NH_4_HCO_3_ buffer at a temperature of 37 °C overnight, resulting in the collection of peptides as filtrate. The digested peptides from each MVs sample underwent desalting on a C18 solid-phase extraction column (Empore^TM^SPE solid-phase extraction column C18 with a diameter layer of 7 mm and volume of 3 mL, Merck, Darmstadt, Germany), concentration via vacuum centrifugation, and reconstitution using 40 µL of 0.1% (*v*/*v*) formic acid.

Twenty micrograms of MVs proteins purified from *E. faecalis* at different pH environments was mixed with 5× loading buffer (0.6 mL 1 mol/L Tris-HCl pH 8.8, 2 mL 10% SDS, 5 mL 50% glycerol, 0.5 mL 2-mercapto ethanol, 1 mL 1% bromophenol blue, and 0.9 mL water), boiled for 5 min, and electrophoresed on a 12.5% polyacrylamide gel at a constant current of 14 mM for 90 min. The protein bands were stained with Coomassie blue R-250.

MVs protein samples produced by *E. faecalis* in pH 7.0 and pH 9.0 environments were added to a C18 reverse chromatography tube (Thermo Scientific Easy Column (Waltham, MA, USA), 10 cm long, 75 μm inner diameter) with added buffer A (0.1% formic acid). The flow rate was maintained at 300 nl/min, and the separation was conducted using a linear gradient in buffer B consisting of 84% acetonitrile and 0.1% formic acid. The TOF Pro mass spectrometer (Bruker, Billerica, MA, USA) operated in positive ion mode with a target intensity set at 1.5 k and a threshold of 2500 for 10 PASEF MS/MS cycles, while active exclusion was enabled with a release time of 0.4 min.

The raw data of mass spectra of *E. faecalis* MVs in different pH environments were combined and searched in the UniProt database (https://www.uniprot.org/, accessed on 30 June 2023, taxon ID: 474186), and MaxQuant 1.5.3.17 software was used for protein identification and quantitative analysis.

The prediction of protein subcellular localization was carried out using the CELLO (http://cello.life.nctu.edu.tw/, accessed on 30 June 2023) classification system. Cluster (http://bonsai.hgc.jp/~mdehoon/software/cluster/software.htm, accessed on 30 June 2023) and Java 3.0 Treeview Software (https://jtreeview.sourceforge.net/, accessed on 30 June 2023) were used for hierarchical cluster analysis. We used the hierarchical cluster algorithm to categorize the proteins that were expressed differently in MVs released by *E. faecalis* in an alkaline environment, and then we presented these differentially expressed proteins in a heatmap. Gene Ontology (GO) (https://geneontology.org/, accessed on 30 June 2023) functional significant enrichment analysis and Kyoto Encyclopedia of Genes and Genomes (KEGG) (https://www.geneontology.org/, accessed on 30 June 2023) pathway significant enrichment analysis were performed on the differentially expressed proteins.

### 2.6. Metabolomics

The MVs samples were thawed slowly at 4 °C, added with a methanol/acetonitrile/water mixture (2:2:1, *v*/*v*), vortexed and sonicated at low temperature for 30 min, placed in the refrigerator at −20 °C for 10 min, centrifuged at 12,000× *g* for 25 min at 4 °C, and the supernatant was vacuum dried for spare parts. For mass spectrometry analysis, the supernatant was redissolved in 150 μL of acetonitrile (acetonitrile/water = 1:1, *v*/*v*), vortexed, and centrifuged at 12,000× *g* for 25 min at 4 °C, and the supernatant was collected for LC-MS analysis.

Next, the supernatants were separated using an Agilent 1290 Infinity LC system at 25 °C. The injection volume of each sample was 2 μL, and the flow rate was 0.5 mL/min. Mobile phase A was water containing 25 Mm ammonium acetate and 25 Mm ammonia, and mobile phase B was acetonitrile. The gradient was set as follows: 0 to 0.5 min: 95% B; 0.5 to 7 min: 95% B to 65% B; 7 to 8 min: 65% B to 40% B; 8 to 9 min: 40% B; 9 to 9.1 min: 40% B to 95% B; and 9.1 to 12 min: 95% B.

The ESI source conditions were set as follows: Ion Source Gas1 (Gas1) as 60, Ion Source Gas2 (Gas2) as 60, curtain gas (CUR) as 30, source temperature: 600 °C, and IonSpray Voltage Floating (ISVF) ± 5500 V. In MS-only acquisition, the instrument was set to acquire over the *m*/*z* range 60–1000 Da, and the accumulation time for the TOF MS scan was set at 0.20 s/spectra. In auto MS/MS acquisition, the instrument was set to acquire over the *m*/*z* range 25–1000 Da, and the accumulation time for product ion scan was set at 0.05 s/spectra. The product ion scan is acquired using information-dependent acquisition (IDA) with high sensitivity mode selected. The parameters were set as follows: the collision energy (CE) was fixed at 35 V with ± 15 eV; declustering potential (DP), 60 V (+) and −60 V (−); exclude isotopes within 4 Da; candidate ions to monitor per cycle: 10.

The raw MS data were converted to MzXML files using ProteoWizard MSConvert before importing into freely available XCMS software (https://xcmsonline.scripps.edu/, accessed on 30 June 2023). For peak picking, the following parameters were used: centWave *m*/*z* = 10 ppm, peakwidth = c (10, 60), prefilter = c (10, 100). For peak grouping, bw = 5, mzwid = 0.025, and minfrac = 0.5 were used. CAMERA (Collection of Algorithms of MEtabolite pRofile Annotation) was used for annotation of isotopes and adducts. In the extracted ion features, only the variables having more than 50% of the nonzero measurement values in at least one group were kept. Compound identification of metabolites was performed by comparing the accuracy of *m*/*z* values (<10 ppm) and MS/MS spectra with an in-house database established with available authentic standards.

After sum-normalization, the processed data were analyzed by R package 4.0.0 (ropls), where it was subjected to multivariate data analysis, including Pareto-scaled principal component analysis (PCA) and orthogonal partial least-squares discriminant analysis (OPLS-DA). The 7-fold cross-validation and response permutation testing were used to evaluate the robustness of the model. The variable importance in the projection (VIP) value of each variable in the OPLS-DA model was calculated to indicate its contribution to the classification. Student’s *t*-test was applied to determine the significance of differences between two groups of independent samples. VIP > 1 and *p* value < 0.05 were used to screen significant changed metabolites. Pearson’s correlation analysis was performed to determine the correlation between two variables.

Group categorization of differential metabolites of *E. faecalis* MVs in an alkaline pH environment, and then GO (https://geneontology.org/, accessed on 30 June 2023) functional significant enrichment analysis and KEGG (https://www.geneontology.org/, accessed on 30 June 2023) pathway significant enrichment analysis were performed on the differentially expressed metabolites.

### 2.7. Differentiation Culture of Human Macrophage In Vitro

The human acute monocytic leukemia cell line (THP-1) was obtained from the National Collection of Authenticated Cell Cultures (NCACC). Cells were routinely cultured in RPMI 1640 medium supplemented with 10% fetal bovine serum (FBS) at 37 °C in a humidified 5% CO_2_ atmosphere. Once THP-1 cells reached confluency between 80 and 90%, cells were stimulated under light-shielded conditions using phorbol-12-myristate-13-acetate (PMA) at a concentration of 50 ng/mL for 48 h to induce macrophage differentiation. After the suspended cells adhered to the wall and differentiated into macrophages, the medium was replaced to wash away non-adherent cells. Then, cells were incubated for 24 h to fully differentiate and obtain dTHP-1 cells. Cytoflex flow cytometry (Beckman, Brea, CA, USA) was used to detect CD68 expression in dTHP-1 cells. Data analysis was performed using FlowJo software 10.0 (https://www.flowjo.com/solutions/flowjo, accessed on 30 June 2023).

### 2.8. Cell Counting Kit-8 (CCK-8) Assay

dTHP-1 cells were plated in a 96-well plate at a concentration of 10^4^ cells per well and left to incubate overnight at 37 °C with 5% CO_2_ in a cell culture incubator. Once the cells adhered to the bottom of the wells, culture medium was replaced, and 10 μL of pH 7.0 MVs (0.5 μg/mL, 1 μg/mL) and pH 9.0 MVs (0.5 μg/mL, 1 μg/mL) were added separately to infect dTHP-1 cells for 2 h before removing the stimulation and continuing cell incubation for another 24 h. PBS was used as a negative control, while LPS at a concentration of 0.5 μg/mL served as a positive control. After infection, dTHP-1 cells were washed three times with PBS, followed by adding 100 μL of fresh culture medium containing CCK-8 reagent per well. The plate was then incubated in darkness for an additional 24 h before measuring absorbance using an enzyme-linked immunosorbent assay reader (Tecan, Männedorf, Switzerland) at a wavelength of 450 nm, and the results could reflect the cell viability.

### 2.9. ELISA

The culture supernatant of dTHP-1 cells that were infected by *E. faecalis* MVs produced under different pH conditions for 2 h was collected. PBS was used as a negative control, while 0.5 μg/mL LPS served as a positive control. The centrifugation of the supernatant was carried out at a speed of 4000 rpm for a duration of 20 min. Subsequently, the levels of interleukin (IL)-6 and tumor necrosis factor (TNF)-α, IL-1β, IL-10, IL-1ra, and transforming growth factor (TGF)-β secreted by dTHP-1 cells were measured using an ELISA kit (Xinbosheng, Shenzhen, China). The absorbance at 450 nm was detected employing an enzyme-linked immunosorbent assay reader (Tecan, Männedorf, Switzerland).

### 2.10. Statistical Analysis

Statistical analyses were performed by GraphPad Prism 8.2.0 (https://www.graphpad.com/, accessed on 1 June 2025). One-way analysis of variance (one-way ANOVA) was used to compare multiple groups, and Bonferroni’s method was used for multiple comparisons. The plate colony counts, BCA protein quantification, CCK-8 results, ELISA results, enzyme labeling instrument readings, and NTA results were compared between groups using a two-tailed *t*-test. A significant difference was considered when *p* < 0.05.

## 3. Results

### 3.1. E. faecalis Releases MVs in Alkaline and Neutral Environments

Following an incubation period of 9 h, the growth of *E. faecalis* reached a plateau at both pH 9.0 and pH 7.0, with the colony count of *E. faecalis* at pH 9.0 remaining consistent with that at pH 7.0 (Figure S1A,B), suggesting that the alkaline environment of pH 9.0 has no significant inhibition effect on the growth of *E. faecalis*. However, our experiment found that *E. faecalis* could not grow under pH 10.0 conditions.

**Figure 1:**
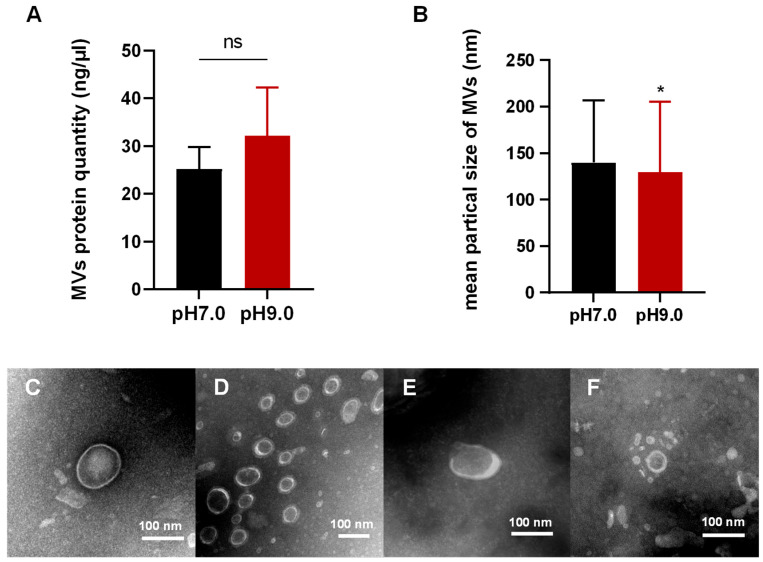
(**A**) BCA protein quantification analysis showed that the alkaline environment pH 9.0 did not alter the production of *E. faecalis* MVs. (**B**) The average particle size of MVs isolated at different pH conditions was examined by NTA, which showed that *E. faecalis* produces smaller sized vesicles in alkaline environment. *n* = 3. The experiment was repeated three times; * *p* < 0.05, ns: non-significant. (**C**–**F**) TEM investigation of MVs isolated at different pH conditions. (**C**) MVs isolated at pH 7.0, magnification ×40.0 K times. (**D**) MVs isolated at pH 7.0, magnification ×30.0 K times. (**E**,**F**) MVs isolated at pH 9.0, magnification ×40.0 K times.

By means of density gradient centrifugation, MVs secreted by *E. faecalis* under pH 7.0 and pH 9.0 conditions were collected, respectively. This experiment shows that MVs can be released by *E. faecalis* in both neutral and alkaline environments. BCA protein quantification analysis indicated no significant difference in the yield of *E. faecalis* MVs between pH 9.0 and pH 7.0 environments (Figure 1A), indicating that the alkaline environment at pH 9.0 does not have a detrimental effect on *E. faecalis* production of MVs.

### 3.2. Morphological Analysis of MVs in E. faecalis

The customary vesicular structure was observed through TEM, which revealed a lipid bilayer structure with a concave cup-shaped nucleus (Figure 1C–F). The diameter of isolated MVs was determined using NTA (Figure 1B). The findings indicated that MVs at pH 7.0 had a particle size of 139.9 ± 67.0 nm, while MVs at pH 9.0 had a particle size of 129.5 ± 75.9 nm. This result suggests that the alkaline environment induced the generation of smaller vesicles by *E. faecalis* (*p* < 0.05).

### 3.3. Proteomic Analysis of MVs

**Figure 2:**
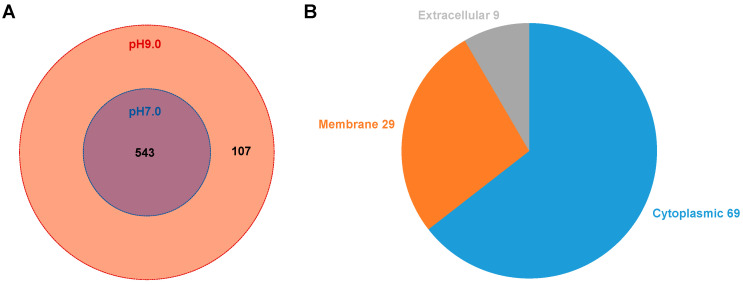
(**A**) Venn diagram of MVs proteins at different pH conditions. (**B**) Subcellular structural localization of *E. faecalis* MVs proteins in alkaline environment by CELLO.

A total of 650 proteins were identified in *E. faecalis* MVs samples, with 650 proteins detected in pH 9.0 MVs, 543 proteins detected in pH 7.0 MVs, and 543 proteins that were detected under both pH conditions (Figure 2A). The top 50 proteins found in MVs under both pH conditions primarily participated in glucose metabolism, cell division, fatty acid metabolism, protein transport, nucleic acid metabolism, mismatch repair, stress response, virulence factors regulation, cell wall formation and hydrolysis processes, translation machinery operation, protein secretion, and lipid metabolism (Table S1).

**Figure 3:**
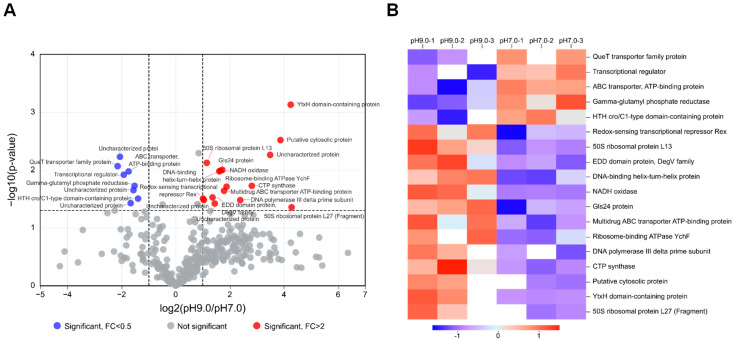
(**A**) Volcano plot of potential differences between MVs isolated at different pH conditions. The red dots represent proteins up-regulated in pH 9.0 MVs, and the blue dots represent proteins down-regulated, and grey represents proteins with no significant difference in expression between pH 7.0 and pH 9.0 environment. (**B**) Cluster analysis heatmap. Each column represents a set of samples (horizontal coordinates are sample information) and each row represents a protein (vertical coordinates are significantly different proteins), where red represents significantly up-regulated proteins, blue represents significantly down-regulated proteins, and white sections represent no protein quantification information.

Fold change (FC) >2.0 (more than 2 times up or less than 0.5 times down) and *p* < 0.05 as expression changes significantly was used to analyze the expression of proteins in *E. faecalis* MVs. In an alkaline environment, we observed up-regulation of 16 proteins and down-regulation of 8 proteins in *E. faecalis* MVs (Figure 3A). Through presence/absence protein screening, we identified 72 proteins specifically expressed in *E. faecalis* MVs under alkaline conditions (Table S2), meeting the criteria of no null values across multiple samples within one group and all data being null in the other group. The hierarchical cluster algorithm was employed to classify differential proteins in pH 9.0 *E. faecalis* MVs, which were visualized using a heatmap (Figure 3B). All the differentially expressed proteins included those involved in glucose metabolism (4 proteins), fatty acid metabolism (6 proteins), cell division (3 proteins), transporters (13 proteins), drug resistance-related functions (3 proteins), nucleic acid metabolism (13 proteins), amino acid metabolism (2 proteins), lipid binding protein function (1 protein), lipid metabolism (2 proteins), stress response pathways (7 proteins), redox processes (2 proteins), virulence factors (2 proteins), transcriptional regulation (7 proteins), mismatch repair mechanisms (1 protein), translation process (4 proteins), and unknown functions (26 proteins). Subcellular structural localization of all differential proteins in pH 9.0 *E. faecalis* MVs was performed (Figure 2B). Specifically, 69 proteins were identified from the cytoplasm, 29 proteins originated from the cell membrane, and 9 proteins were derived externally. Notably, the majority of *E. faecalis* MVs proteins are predominantly localized in both the cytoplasm and cytoplasmic membrane.

**Figure 4:**
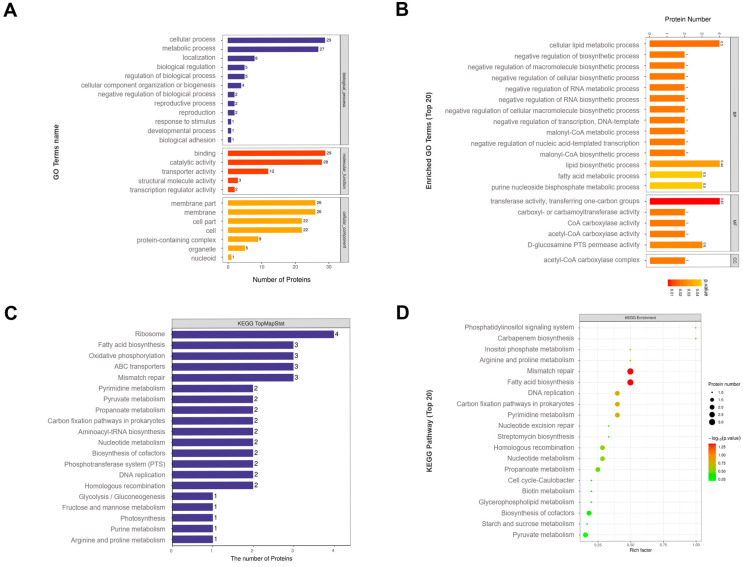
(**A**) GO classification of differential proteins in pH 9.0 *E. faecalis* MVs. (**B**) GO enrichment pathway for differential proteins in pH 9.0 *E. faecalis* MVs. (**C**) KEGG pathway annotation map for differential proteins in pH 9.0 *E. faecalis* MVs. (**D**) KEGG pathway enrichment bubble map of differential proteins in pH 9.0 *E. faecalis* MVs.

The GO classification showed that the proteins in pH 9.0 *E. faecalis* MVs have been found to be differentially affected in biological processes such as cellular process, metabolic process, cellular component, cell membrane and cell, molecular function, binding, and catalytic activity, and so on (Figure 4A).

According to GO functional analysis (Figure 4B), the differential proteins in pH 9.0 *E. faecalis* MVs exhibit negative regulation of cellular lipid metabolic process and polymer biosynthesis process (macromolecule biosynthetic process), negative control of RNA biosynthesis, negative regulation of transcription, DNA template activity (DNA-templated), malonyl CoA biosynthetic process, and other important biological processes. They also possess molecular functions such as transferase activity for transferring one-carbon groups, acetyl-CoA carboxylase activity, CoA carboxylase activity, carboxyl or carbamoyl transferase activity, and D-glucosamine phosphatase transfer system (PTS) permease activity. Furthermore, the differential proteins in pH 9.0 *E. faecalis* MVs also have a localization function like that of the acetyl-CoA carboxylase complex.

The KEGG pathway classification (Figure 4C,D) results revealed significant expression alterations in ribosome, fatty acid biosynthesis, oxidative phosphorylation, ABC transporters, mismatch repair, and other pathways in *E. faecalis* MVs under alkaline conditions.

### 3.4. Metabolomic Analysis of MVs

The stability of the instrument, the reproducibility of the experiment, and the reliability of the data quality were comprehensively evaluated by using six quality control contents: total ion flow diagram of QC samples, principal component analysis of overall samples, correlation of QC samples, Hotelling’s T2 test of overall samples, multivariate control chart of QC samples, and relative standard deviation of QC samples. The results showed that the stability of the instrumental analysis system was good, and the experimental data were stable and reliable; the differences in metabolic profiles obtained in the experiment could reflect the biological differences between the samples (Figure S2A–L).

**Figure 5:**
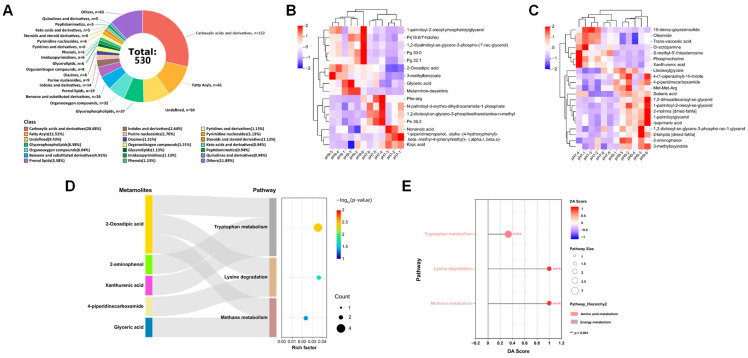
(**A**) The quantification and proportional distribution of metabolites in *E. faecalis* MVs identified under the conditions of pH 7.0 and pH 9.0 across various chemical classification categories were analyzed. Metabolites lacking an identifiable chemical classification were categorized as “undefined”. Meanwhile, those chemical classification subsets harboring fewer than 10 metabolites were pooled and collectively denoted as “others”. (**B**,**C**) Heatmap of cluster analysis for DEMs of *E. faecalis* MVs in negative ion mode (**B**) and positive ion mode (**C**) under pH 9.0 conditions. In the graphical representation, each column is congruent to a particular group of samples, and every row symbolizes a single metabolite. Notably, within the context of the heatmap, the red hue is indicative of metabolites that have undergone significant up-regulation, whereas the blue shade denotes those metabolites with pronounced down-regulation. (**D**) Sankey bubble diagram of KEGG enrichment pathways for DEMs in *E. faecalis* MVs under pH 9.0 condition. The sankey diagram positioned on the left illustrates the specific enrichment pathways to which the DEMs pertain. Meanwhile, the bubble diagram situated on the right exhibits the Rich ratio (plotted on the X-axis), enrichment items (displayed on the Y-axis), the quantity of metabolites (indicated by the bubble size), and the *p*-value (depicted by the color). (**E**) Lollipop chart of KEGG enrichment pathways for DEMs in *E. faecalis* MVs under pH 9.0 condition. The Y-axis denotes the names of the pathways, while the X-axis signifies the differential abundance score (DA score). The DA score represents the comprehensive total variation of all metabolites within the pathway. When the score exceeds 0, it implies that the expression trends of all the identified metabolites in this pathway are up-regulated. Conversely, when the score is below 0, it indicates that the expression trends of all the identified metabolites in this pathway are down-regulated. The length of each line segment corresponds to the absolute value of the DA score. The size of the dot at the terminal end of the line segment reflects the quantity of metabolites in the pathway. Moreover, the darkness of the dot’s color is directly proportional to the magnitude of the DA score value. *** *p* < 0.001.

A total of 530 metabolites were detected in *E. faecalis* MVs after merging the positive and negative ion modes, with 311 metabolites identified in the positive ion mode and 219 metabolites identified in the negative ion mode. The major identified metabolites encompass carboxylic acids and derivatives, fatty acyls, prenol lipids, glycerophospholipids, organooxygen compounds, benzene and substituted derivatives, prenol lipids, and indoles and derivatives (Figure 5A).

Among the shared metabolites in *E. faecalis* MVs under two pH conditions (Table S3), the primarily involved are prenol lipids, steroids and steroid derivatives, organooxygen compounds, benzene and substituted derivatives, organonitrogen compounds, fatty acyls, carboxylic acids and derivatives, and peptidomimetics.

Metabolites in *E. faecalis* MVs with significant differences under pH 9.0 condition were identified using OPLS-DA VIP values >1 and *p* < 0.05 (Table S4). The differential metabolites (DEMs) in *E. faecalis* MVs under pH 9.0 condition belonged to various categories, such as glycerophospholipids (10 metabolites), pyridines (2 metabolites), keto acids and derivatives (1 metabolite), benzene and substituted derivatives (2 metabolites), organooxygen compounds (1 metabolite), organonitrogen compounds (1 metabolite), pyrans (1 metabolite), triazines (1 metabolite), sphingolipids (1 metabolite), fatty acyls (4 metabolites), carboxylic acids and derivatives (3 metabolites), prenol lipids (1 metabolite), glycerophospholipids (1 metabolite), lactones (1 metabolite), indoles and derivatives (1 metabolite), diazinanes (1 metabolite), phenols (1 metabolite), nucleosides, nucleotides, and analogues (1 metabolite), quinolines and derivatives (1 metabolite), as well as undefined metabolites (2 metabolites). A total of 23 up-regulated and 14 down-regulated metabolic changes were observed in *E. faecalis* MVs under pH 9.0 conditions.

The hierarchical cluster algorithm was employed to cluster and classify the EDMs in *E. faecalis* MVs under a pH 9.0 environment. The results were visualized as a heatmap (Figure 5B,C).

The KEGG pathway enrichment analysis (Figure 5D) demonstrated notable alterations in essential pathways, including tryptophan metabolism, lysine degradation, and methane metabolism, in *E. faecalis* MVs at pH 9.0, and the metabolite expression of the above pathways showed an upward trend (Figure 5E). An integrative multi-omics analysis, combining proteomics, revealed no overlap between the KEGG pathways enriched in the current study and those identified in previous protein profiling analyses.

### 3.5. The Effect of E. faecalis MVs on dTHP-1 Inflammatory Response Regulation

The THP-1 cells were observed as a circular suspension under a microscope (Figure S3A). Upon PMA induction, the differentiated dTHP-1 cells exhibited irregular adherent growth with visible pseudopodia (Figure S3B). Flow cytometry analysis revealed that the specific marker CD68 was expressed in dTHP-1 cells but not in THP-1 cells, indicating successful differentiation of THP-1 cells into dTHP-1 cells by PMA induction. CCK-8 results showed that both pH 7.0 and pH 9.0 *E. faecalis* MVs at a concentration of 1 μg/mL inhibited the viability of dTHP-1 cells, with no statistical difference between the two pH levels (Figure S4). However, 0.5 μg/mL *E. faecalis* MVs had no effect on the metabolic activity of dTHP-1 cells under both pH conditions. These findings suggest that the inhibitory effect of *E. faecalis* MVs on the viability of dTHP-1 cells depended on dosage and was unaffected by the pH environment. 

**Figure 6:**
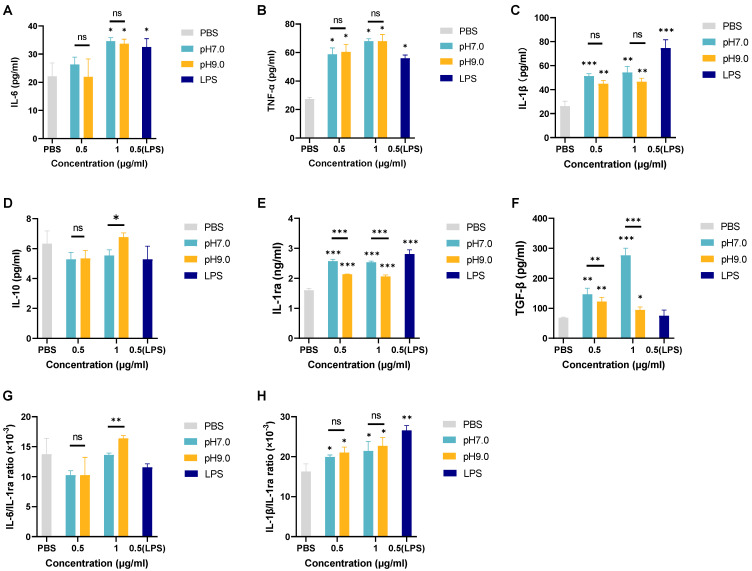
Effects of *E. faecalis* MVs on the secretion of IL-6 (**A**), TNF-α (**B**), IL-1β (**C**), IL-10 (**D**), IL-1ra (**E**), TGF-β (**F**), IL-6/IL-1ra ratio (**G**), and IL-1β/IL-1ra ratio (**H**) by dTHP-1 cells under different pH conditions. n = 3. The experiment was repeated three times; * *p* < 0.05, ** *p* < 0.01, *** *p* < 0.001, ns: non-significant.

The ELISA results (Figure 6A–F) showed that *E. faecalis* MVs at pH 7.0 and pH 9.0 significantly promoted the secretion of the inflammatory factors IL-6, TNF-α, IL-1β, and IL-1ra by macrophages. IL-6 secretion was promoted in a dose-dependent manner, while the IL-1β/IL-1ra ratio significantly increased and the IL-6/IL-1ra ratio showed no significant change. *E. faecalis* MVs had no significant effect on the secretion of the anti-inflammatory factor IL-10. Additionally, *E. faecalis* MVs promoted the secretion of the regulatory factor TGF-β in macrophages. The promoting effect of pH 9.0 *E. faecalis* MVs on TGF-β was significantly weaker than that of pH 7.0 *E. faecalis* MVs.

## 4. Discussion

This study presents novel insights into how *E. faecalis* modulates its MVs proteome and metabolome under alkaline stress conditions, contributing to our understanding of its persistence in endodontic environments. The data align with several pieces of evidence that bacterial adaptation to environmental stress involves fine-tuned metabolic regulation, often mediated by MVs.

OG1RF ATCC 47077 is a strain of *E. faecalis* that is commonly utilized in endodontic studies. It is a well-characterized laboratory strain of *E. faecalis* that was originally isolated from a human clinical infection and expresses gelE and sprE, which encode gelatinases and serine proteases with a high degree of biofilm formation.

Evans et al. demonstrated that *E. faecalis* JH2-2 could survive at pH values below 11.5 [36]. This finding further elucidated the prevalence of *E. faecalis* in root canals following treatment failure. Additionally, the study revealed that *E. faecalis* could directly form biofilms on Ca(OH)_2_ paste within root canals. It was reported that the cultivation of *E. faecalis* ATCC 33186 under pH 10.0 displayed a significant reduction in protein synthesis [37]. In this study, two alkaline environments with pH 9.0 and pH 10.0 were designed. However, it was observed that *E. faecalis* OG1RF ATCC 47077 exhibited minimal growth under pH 10.0 conditions while remaining unaffected at pH 9.0. Thus, pH 9.0 was chosen as the alkaline environment to analyze the changes in the proteomic profiles of *E. faecalis* MVs and its immune-regulatory effect on macrophages.

Previous studies have confirmed that G^+^ bacteria, such as *L. monocytogenes*, *Streptococcus pneumonia* (*S. pneumonia*), *E. faecalis*, and *S. aureus*, are capable of producing MVs despite their thicker cell wall [38,39,40,41]. The release mechanism of MVs in G^+^ bacteria has been hypothesized to occur through passive budding due to inflation pressure, enzymatic modification of the cell wall leading to pore expansion, or deformation of the cell wall and MVs passing through channels smaller than their diameter [23]. This study confirmed that *E. faecalis* is capable of releasing MVs with diameters similar to those of porcine-derived *E. faecalis* CQ20 MVs [27]. Furthermore, autolysin detected in *E. faecalis* MVs suggests that *E. faecalis* might release MVs through autolysis of its cell wall.

In this study, a total of 543 *E. faecalis* MVs proteins associated with biofilm formation, virulence factors, and resistance to stress environments were identified under both pH conditions. In *E. faecalis* MVs, Gel, an important virulence factor for *E. faecalis*, was detected, which could hydrolyze host proteins and collagen, facilitating the invasion of *E. faecalis* into dentinal tubules [42,43,44]. The folded protein PrsA was also found to exist in MVs of *E. faecalis* under both pH conditions. PrsA was found to be involved in the final successful folding of group A *Streptococcus* (GAS) virulence factor SpeB and *E. faecalis* virulence factor Gel [45,46]. Furthermore, other studies conducted by our research group indicated that the *ΔprsA* mutant strain of *Streptococcus mutans* (*S. mutans*) exhibits increased cell surface hydrophobicity and reduced biofilm formation ability, suggesting a potential role for PrsA in bacterial biofilm development [47]. Additionally, the presence of chaperone proteins DnaK and DnaJ in *E. faecalis* MVs was identified, which aids in maintaining proper protein conformation during stress conditions such as high temperature and high salt [48]. Studies have shown that the DnaK/DnaJ system is significantly up-regulated in *Mycobacterium tuberculosis* (*M. tuberculosis*) and *Salmonella* under environmental stress conditions, helping the bacteria to survive [49]. It has been observed that heat shock, high salt concentration, SDS exposure, and H_2_O_2_ can induce transcription of the *dnaK* and *dnaJ*, indicating their role in assisting *E. faecalis* resistance against the stress environment of treated root canals [50,51,52,53].

This study revealed that *E. faecalis* MVs production remained unchanged under pH 9.0 conditions compared to pH 7.0 but exhibited a smaller diameter. Additionally, pH 9.0 *E. faecalis* MVs expressed 72 specific proteins associated with alkaline resistance, drug resistance, stress-associated proteins, and virulence factors. Subcellular structural localization of all differential proteins was found to originate from the cytoplasm, cell membrane, and cell surface. *E. faecalis* is able to counteract the highly alkaline environment by synthesizing ATP synthase to facilitate proton influx into the cell. Some studies have found that the stress environment induces up-regulation of ATP synthase expression [54,55,56]. After pretreating *E. faecalis* with a proton pump inhibitor and exposing it to alkaline pH for 30 min, the survival rate of *E. faecalis* decreased significantly [57], indicating a potential correlation between the alkaline resistance of *E. faecalis* and its proton pump activity. In our study, specific expression of δ and β subunits of ATP synthase was identified in MVs derived from alkaline-adapted *E. faecalis*, providing further evidence for its alkali tolerance. A variety of stress proteins, including the RelA_SpoT domain protein (C7D128), Gls24 general stress protein (C7D128), and YtxH domain protein (A0A8B3RRY2), were up-regulated or exclusively expressed in pH 9.0 *E. faecalis* MVs. Notably, the presence of RelA_SpoT domain proteins was only observed in MVs derived from *E. faecalis* in an alkaline environment. Previous studies have confirmed that RelA and SpoT function as ppGpp synthetases, with ppGpp serving as a key regulatory factor for bacterial virulence and resistance against environmental stresses [58,59,60,61,62,63]. However, further experimental verification is required to determine if *E. faecalis* generates ppGpp through the catalytic activity of RelA and SpoT in response to an alkaline environment. The adaptation of *E. faecalis* to alkaline environments, as seen in the selective expression of stress-related proteins and ATP synthase subunits within MVs, highlights a complex regulatory network that sustains cellular homeostasis. Similar metabolic plasticity has been observed in *Streptococcus thermophiles* (*S. thermophiles*), where CO_2_ metabolism plays a central role in supporting amino acid and nucleotide biosynthesis, especially under nutrient-limited conditions. Arioli et al. [64] demonstrated that CO_2_ limitation induces auxotrophy for arginine, aspartate, and uracil and alters the expression of key metabolic enzymes, directly impacting bacterial growth and viability. These findings underline the broader relevance of environmental modulation of central metabolism in G^+^ bacteria. They suggest that *E. faecalis* may leverage similar strategies to thrive in high-pH and osmotically stressful niches via its MV-mediated adaptation mechanisms.

In this study, betaine and choline were detected in both alkaline and neutral environment *E. faecalis* MVs. Betaine is widely found in animals, plants, and microorganisms and is an important protective agent for most microorganisms against drought and hypertonic environments, as well as balancing intra- and extracellular osmotic pressure and acting as a stabilizer of intracellular enzymes [65,66]. Choline, a precursor substance of betaine, is similarly associated with bacterial osmoprotection [66]. Rinsing agents such as NaCl and NaClO used in RCT enter the dentin tubules to regulate their osmotic pressure, creating a high-salt environment. Betaine in *E. faecalis* MVs may favor the survival of *E. faecalis* in high-salt root canal environments. In addition, desferrioxamine (DFO) was detected in *E. faecalis* MVs. DFO is a chelator of iron ions, and it has been shown that DFO binds free intracellular iron ions and resists DNA damage caused by H_2_O_2_ [67,68]. When *E. faecalis* was cultured in an anaerobic environment and treated with H_2_O_2_ for 20 min, it was found that the survival rate of *E. faecalis* treated with iron chelator was much higher than that of the untreated group [69], which could also explain the high detection rate of *E. faecalis* in RAP. Antibiotics have also been found in *E. faecalis* MVs, including lincomycin, valinomycin, apramycin, clarithromycin, erythromycin, and so on. Our previous proteomic analysis of *E. faecalis* MVs also revealed the presence of multidrug transporter proteins in MVs under different pH conditions (Tables S1 and S2), and we hypothesized that the active transport capacity of *E. faecalis* for lincomycin and clarithromycin may be related to its MVs, either through direct release from MVs or through the transporter of multidrug transporter proteins.

Kojic acid was down-regulated 0.566-fold in alkaline *E. faecalis* MVs. Li et al. found that kojic acid inhibited the biofilm formation of *Acinetobacter baumannii* (*A. baumannii*), with the most pronounced inhibitory effect at concentrations above 3 mM [70]. In addition, kojic acid inhibits the formation of bacterial biofilms of *L. monocytogenes*, *B. subtilis*, *E. coli*, etc. [71]. However, the effect of kojic acid on the biofilm-forming capacity of *E. faecalis* remains to be investigated.

Our study showed that both lysine degradation and tryptophan metabolism pathways were significantly up-regulated in pH 9.0 *E. faecalis* MVs. 2-Oxoadipic acid, an intermediate product of lysine and tryptophan degradation [72], was up-regulated 1.428-fold in alkaline *E. faecalis* MVs, suggesting that lysine and tryptophan degradation of *E. faecalis* is active in an alkaline environment and that acetyl-coenzyme A generated in this process is involved in fatty acid biosynthesis. This is consistent with the results of proteomic studies of MVs, in which we found that both the α and β subunits (AccA and AccD) of acetyl coenzyme A carboxylase were specifically expressed only in pH 9.0 *E. faecalis* MVs (Table S2).

The overlap of significantly different pathways in metabolomics and proteomics in this study was not obvious, and the low percentage of significantly different metabolites made it difficult to integrate and analyze significantly different pathways in proteins and metabolites. Considering that *E. faecalis* is extremely alkaline tolerant, the alkaline stress stimulus used in this experiment may be insufficient, and subsequent experiments may continue to explore the mechanism of alkaline tolerance of *E. faecalis* MVs by increasing the pH of the alkaline medium. In this experiment, it was observed that *E. faecalis* MVs contain a large number of antibiotics and hypertonicity-resistant and H_2_O_2_-resistant metabolites, but the mechanism of action has not been explored in depth. The study can be followed up by exposing *E. faecalis* to lincomycin, hypersaline medium, or H_2_O_2_, and exploring the role of lincomycin, hypertonicity-tolerant, and H_2_O_2_-tolerant metabolites in its MVs by targeting metabolomics to detect if there are any significant differences in these metabolites from the untreated *E. faecalis* MVs. Differences in biofilm-forming capacity of *E. faecalis* can also be observed by treating *E. faecalis* with tretinoin versus the untreated group.

Macrophages are among the earliest immune cells to migrate into periapical tissues during the development of periapical inflammation [73,74,75]. However, previous studies have primarily focused on the effects of *E. faecalis* on macrophage proliferation. Some studies have demonstrated that *E. faecalis* could induce necrotizing apoptosis in macrophages [76,77]. Nevertheless, the role of *E. faecalis* MVs in this aspect requires further exploration. Some studies have found that MVs can be endocytosed by macrophages, thereby reducing phagocytosis, NF-κB activation, cell initiation, and strong pro-inflammatory responses, including IL-1β secretion and activation of cell death via inflammasome activity [78,79]. Other studies have shown that MVs from *Porphyromonas gingivalis* (*P. gingivalis*) and *Rhodococcus equi* (*R. equi*) induced macrophage pyroptosis, with *P. gingivalis* MVs exhibiting stronger induction ability than *P. gingivalis* itself [80,81]. Our study revealed that *E. faecalis* MVs under neutral and alkaline pH conditions possessed the ability to inhibit macrophage viability. However, further research is needed to elucidate the specific mechanism and explore related signaling pathways.

*E. faecalis* MVs were found to significantly promote the secretion of the pro-inflammatory cytokines IL-6, TNF-α, and IL-1β by macrophages. However, the promotional effect of MVs did not differ significantly under neutral and alkaline conditions. *E. faecalis* MVs also promote the secretion of the anti-inflammatory cytokine IL-1ra, but in comparison with the control group treated with PBS, the IL-1β/IL-1ra ratio is increased significantly after *E. faecalis* MVs treatment of macrophages. Studies [82,83,84] have shown that the IL-1β/IL-1ra ratio is a core indicator of inflammatory regulation; its elevation typically indicates a pro-inflammatory state associated with disease severity and poor prognosis. *E. faecalis* MVs promote the secretion of the regulatory factor TGF-β by macrophages. Compared to pH 7.0, the promoting effect of pH 9.0 *E. faecalis* MVs is significantly weaker. Although TGF-β is traditionally considered anti-inflammatory [85,86], an increasing number of studies have demonstrated its pro-inflammatory effects. Researches [87,88] have found that a high-glucose environment or the synergistic action of TLR4 with TGF-β can increase the expression of pro-inflammatory markers (e.g., TSP-4, IL-6, and MCP-1) in macrophages. This indicates that TGF-β has a pro-inflammatory effect in certain inflammatory pathways. In models of myocardial infarction and kidney injury, elevated TGF-β levels promote inflammatory responses [89,90]. In summary, *E. faecalis* MVs exhibit significant pro-inflammatory effects; however, there is no significant difference in pro-inflammatory effects between MVs under different pH conditions. The differential proteins identified in *E. faecalis* MVs under different pH conditions mainly belonged to metabolism-related proteins, stress proteins, and drug resistance-related proteins. KEGG pathway analysis also revealed significant changes in fatty acid metabolism and mismatch repair pathways in the alkaline *E. faecalis* MVs. Moreover, the viability inhibitory effects of *E. faecalis* MVs on dTHP-1 cells were not enhanced by the alkaline environment. One possible explanation is that under an alkaline environment, *E. faecalis* tends to enhance its environmental adaptation ability through increased release of MVs rather than its pathogenicity. However, further investigation is still required to support this hypothesis.

## 5. Conclusions

We verified that *E. faecalis* produces MVs in both alkaline and neutral environments and that the protein composition and metabolic activity within the MVs of *E. faecalis* differed under different pH conditions. The differential proteins ATP synthase and stress proteins, as well as the differential metabolic pathways lysine such as degradation and the tryptophan metabolism pathway, were enriched in pH 9.0 *E. faecalis* MVs and may be associated with the alkaline tolerance of *E. faecalis*. We also found that *E. faecalis* MVs dose-dependently inhibited the viability of macrophages. Additionally, *E. faecalis* MVs promote the secretion of IL-6, TNF-α, and IL-1β by macrophages and increase the IL-1β/IL-1ra ratio in macrophages, indicating the pro-inflammatory properties of *E. faecalis* MVs on macrophages. Our results will provide a basis for elucidating the pathogenic mechanism of *E. faecalis* persistent infection in PAP and provide new ideas and targets for the prevention and treatment of PAP.

## Data Availability

The mass spectrometry proteomic raw data have been deposited to the ProteomeXchange Consortium (https://proteomecentral.proteomexchange.org, accessed on 11 October 2024) via the iProX partner repository with the dataset identifier PXD056708.

## Supplementary Materials

The following supporting information can be downloaded at: https://www.mdpi.com/article/10.3390/microorganisms13061344/s1, Figure S1: The growth of *E. faecalis* under various pH conditions; Figure S2: Quality assessment of metabolomics experimental data; Figure S3: Morphological observation of THP-1 and dTHP-1 cells; Figure S4: Effects of *E. faecalis* MVs on dTHP-1 cell viability under different pH conditions; Table S1: Top 50 common proteins shared by MVs under pH 7.0 and pH 9.0 conditions; Table S2: Classification of major functions of differential proteins in *E. faecalis* pH 9.0 MVs; Table S3: Common metabolites shared by MVs under pH 7.0 and pH 9.0 conditions; Table S4: Significantly different metabolites of *E. faecalis* EVs at pH 9.0. Excel table: Metabolites identified in positive and negative ion mode, along with their chemical classification information.
