# Supplementary material for: Membrane Vesicles of Enterococcus faecalis: In Vitro Composition Analysis and Macrophage Inflammatory Response Under Different pH Conditions
Source: Microorganisms. 2025 Jun 10;13(6):1344. doi: 10.3390/microorganisms13061344 (PMC12195402; doi:10.3390/microorganisms13061344)
Supplement: Supplementary file 1 [file microorganisms-13-01344-s001.zip › Supplementary Material.pdf]

Table S1 Top 50 common proteins shared by MVs under pH 7.0 and pH 9.0 conditions

Function	Protein	gene	Protein name
glucose metabolism	E6ER18	*eno*	Enolase
glucose metabolism	P23530	*ptsI*	Phosphoenolpyruvate-protein phosphotransferase
glucose metabolism	Q833J0	*tpiA*	Triosephosphate isomerase
glucose metabolism	Q836R3	*pfkA*	ATP-dependent 6-phosphofructokinase
cell division	O07111	*ftsA*	Cell division protein FtsA
cell division	Q836V4	*ftsZ*	Cell division protein FtsZ
fatty acid metabolism	X5NU53	*fabZ*	3-hydroxyacyl-[acyl-carrier-protein] dehydratase FabZ
protein transport	Q832R5	EF_2153	Putative ABC transporter ATP-binding protein EF_2153
protein transport	X5NZ58	*secA*	Protein translocase subunit SecASecA
protein transport	Q834B4	*pstB1*	Phosphate import ATP-binding protein PstB 1
protein transport	Q834B3	*pstB2*	Phosphate import ATP-binding protein PstB 2
protein transport	Q839D4	*ecfA2*	Energy-coupling factor transporter ATP-binding protein EcfA2
protein transport	X5NWX7	P746_00557	Cobalt transport ATP-binding protein cbiO
nucleic acid metabolism	E0HFD7	*rpoD*	RNA polymerase sigma factor SigA

nucleic acid metabolism	S4D127	*rpoB*	DNA-directed RNA polymerase subunit beta
nucleic acid metabolism	Q82Z99	*rny*	Ribonuclease Y
nucleic acid metabolism	V7ZJS7	*rpoC*	DNA-directed RNA polymerase subunit beta
mismatch repair	A0A2I4PDG1	*recA*	Protein RecA (Fragment)
mismatch repair	A0A4U3LKV2	*mutS*	DNA mismatch repair protein MutS (Fragment)
mismatch repair	Q834D0	*nfo*	Probable endonuclease 4
stress response	P37062	*npr*	NADH peroxidase
stress response	S4E739	*clpB*	Chaperone protein ClpB
stress response	D4ETY7	*dnaJ*	Chaperone protein DnaJ
stress response	Q835R7	*dnaK*	Chaperone protein DnaK
virulence factors	Q833V7	*gelE*	GelatinaseE
virulence factors	A0A059N3T7	HMPREF2097_00972	Serine protease
cell wall formation	A0A826HE79	*murG*	UDP-N-acetylglucosamine--N-acetylmuramyl-(pentapeptide) pyrophosphoryl-undecaprenol N-acetylglucosamine transferase
cell wall formation	A0A8F5MVW7	KVY10_06030	LysM peptidoglycan-binding domain-containing protein
cell wall formation	A0A059N1U7	*mltG*	Endolytic murein transglycosylase
cell wall formation	A0A4U3MSP2	EY666_00305	LTA synthase family

			protein (Fragment)
cell wall hydrolysis processes	X5NXG6	P746_00032	Peptidoglycan hydrolase
cell wall hydrolysis processes	A0A142BWZ2	*salB*	Serine protease (Fragment)
translation process	X5NU17	*valS*	Valine--tRNA ligase
translation process	E6ELF6	*tyrS1*	Tyrosine--tRNA ligase 1
translation process	X5NTX6	*thrS*	Threonine--tRNA ligase
translation process	A0A4U3LT08	*potA*	Spermidine/putrescine import ATP-binding protein PotA (Fragment)
translation process	X5NWQ2	*argS*	Arginine--tRNA ligase
translation process	E6IBW2	*rpsB*	30S ribosomal protein S2
translation process	Q831V0	*tsf*	Elongation factor Ts
translation process	D4EVD7	*lepA*	Elongation factor 4
translation process	A0A0M2AAN4	*asnS*	Asparagine--tRNA ligase
translation process	V7ZUC5	*rlmN*	Probable dual-specificity RNA methyltransferase RlmN
translation process	Q834T4	*der*	GTPase Der
translation process	C7D3N3	*obg*	GTPase Obg
translation process	V7ZKX7	*infB*	Translation initiation factor IF-2
translation process	V7ZPX5	T481_09880	Peptide chain release factor 3 (Fragment)
protein secretion	P37710	EF_0799	Autolysin
protein secretion	C7D342	*lspA*	Lipoprotein signal peptidase
protein secretion	X5NX70	*prsA*	Foldase protein PrsA
lipid metabolism	V7ZUB7	*ackA*	Acetate kinase

Table S2 Classification of major functions of differential proteins in *E. faecalis* pH 9.0 MVs

Function	Protein	gene	Protein name	Fold change
glucose metabolism	A0A1B4XKC2	*manX*	EIIAB-Man	—
glucose metabolism	A0A3N3YYR6	EGW70_14115	PTS beta-glucoside transporter subunit EIIBCA	—
glucose metabolism	A0A125W2X5	HMPREF9498_02779	PTS system, beta-glucoside-specific, IIABC component	—
glucose metabolism	A0A4V5UW79	*ascB*	6-phospho-beta-glucosidase (Fragment)	—
cell division	X5NUM3	*divIB*	Cell division protein DivIB	—
cell division	Q836V4	*sepF*	Cell division protein SepF	—
cell division	V7ZR55	T481_03185	Cell division protein FtsW	
fatty acid metabolism	X5NVN6	*accD*	Acetyl-coenzyme A carboxylase carboxyl transferase subunit beta	—
fatty acid metabolism	X5NVC9	*accA*	Acetyl-coenzyme A carboxylase carboxyl transferase subunit alpha	—
fatty acid metabolism	A0A1B4XKD8	*proA*	Gamma-glutamyl phosphate reductase	0.349
fatty acid metabolism	X5NVD5	P746_01820	3-oxoacyl-[acyl-carrier-protein] synthase 2	—
fatty acid metabolism	A0A4V5V013	EY666_03055	(3R)-hydroxymyristoyl-[acyl-carrier-protein] dehydratase (Fragment)	—
fatty acid metabolism	A0A4U3L8E2	*pgsA*	CDP-diacylglycerol--glycerol-3-phosphate 3-phosphatidyltransferase	—

			(Fragment)	
protein transport	Q834X7	*atpD*	V-type ATPase subunit D	—
protein transport	Q831A2	*atpH*	ATP synthase subunit delta	—
protein transport	V7ZPN6	T481_10790	V-type ATPase subunit F	—
protein transport	A0A4U3KLN0	EY666_18195	QueT transporter family protein	0.226
protein transport	S4EGW2	D920_01023	ABC transporter, ATP-binding protein	0.300
protein transport	C7D4R1	EFBG_01494	Mn^{2+}/Fe^{2+} transporter	—
protein transport	D4ESV3	HMPREF9377_00609	Sodium/glutamate symporter	—
protein transport	A0A059N1A9	HMPREF2097_01789	Glutamine ABC transporter, ATP-binding protein GlnQ	—
protein transport	X5NUF5	P746_01497	Arginine/ornithine antiporter	—
protein transport	V7ZP78	T481_05975	Potassium transporter Trk	—
protein transport	V7ZPG6	T481_07340	Cation transporter	—
protein transport	V7ZJT7	T481_17480	Peptide ABC transporter substrate-binding protein	—
protein transport	U6S6D3	D350_00352	Putative permease	—
drug resistance	V7ZQ40	T481_08815	Multidrug MFS transporter	—
drug resistance	V7ZPC8	T481_07745	Multidrug transporter	—
drug resistance	V7ZNX0	T481_09810	Multidrug ABC transporter ATP-binding protein	3.451
nucleic acid metabolism	A0A4V5UYT0	EY666_08655	50S ribosomal protein L27 (Fragment)	19.461
nucleic acid metabolism	C7CYS2	*rplM*	50S ribosomal protein L13	2.220
nucleic acid metabolism	X5NYB7	*upp*	Uracil phosphoribosyltransferase	—

nucleic acid metabolism	Q836G5	*pyrG*	CTP synthase	7.026
nucleic acid metabolism	V7ZMT4	*dnaX*	DNA polymerase III subunit gamma/tau	—
nucleic acid metabolism	R3H6E1	WOK_00580	HTH cro/C1-type domain-containing protein	0.382
nucleic acid metabolism	A0A059N0R9	HMPREF2097_01872	DNA-binding helix-turn-helix protein	3.051
nucleic acid metabolism	Q6WS12	—	RNA methyl transferase-like	—
nucleic acid metabolism	Q839V5	*cysS*	Cysteine--tRNA ligase	—
nucleic acid metabolism	A0A4U3M6A3	EY666_11110	DNA topoisomerase (ATP-hydrolyzing) (Fragment)	—
nucleic acid metabolism	Q839Z1	*gyrB*	DNA gyrase subunit B	—
nucleic acid metabolism	A0A8D9MME0	*rep*	ATP-dependent DNA helicase	—
nucleic acid metabolism	A0A059N2Q5	HMPREF2097_01566	Nucleic acid-binding domain protein	—
amino acid metabolism	D4EY17	HMPREF9377_02443	M42 glutamyl aminopeptidase	—
amino acid metabolism	X5NYM8	P746_00467	Aminoacyl-histidine dipeptidase	—
lipid binding	A0A059N0K2	HMPREF2097_01858	EDD domain protein, DegV family	2.570
lipid metabolism	A0A4U3KYV6	EY666_15730	Phosphatase PAP2 family protein (Fragment)	—
lipid metabolism	A0A3N3S1K2	EGW70_06040	Inositol monophosphatase	—

			family protein	
stress response	V7ZS97	*ychF*	Ribosome-binding ATPase YchF	3.685
stress response	E0HAQ4	*rex*	Redox-sensing transcriptional repressor Rex	2.015
stress response	C7D128	EFBG_00211	RelA_SpoT domain-containing protein	—
stress response	A0A059MW82	HMPREF2097_03531	Universal stress family protein	—
stress response	O32593	*gls24*	Gls24 protein	3.307
stress response	A0A8B3RRY2	EU507_11430	YtxH domain-containing protein	18.999
stress response	A0A4U3MDJ6	EY666_06900	Universal stress protein (Fragment)	—
oxidoreductase	A0A828QRJ4	*nox*	NADH oxidase	3.150
oxidoreductase	A0A059N4D6	HMPREF2097_00705	Oxidoreductase, short chain dehydrogenase/reductase family protein	—
virulence factors	E1AS09	*efaA*	Endocarditis specific antigen (Fragment)	—
virulence factors	F6KLM4	pLG2-0070	PspC domain-containing protein	—
transcriptional regulation	X5NY50	*codY*	GTP-sensing transcriptional pleiotropic repressor CodY	—
transcriptional regulation	V7ZPP0	*ctsR*	Transcriptional regulator CtsR	—
transcriptional regulation	X5NXB2	P746_01178	Transcriptional regulator, TetR family protein	—
transcriptional	X5NY33	P746_00806	Transcription antiterminator,	—

regulation			BglG family protein	
transcriptional regulation	V7ZKV7	T481_15730	Transcriptional regulator	0.265
transcriptional regulation	X5NUG1	P746_01480	Trans-acting positive regulator	—
transcriptional regulation	X5NXQ0	P746_00359	Transcriptional activator rinA	—
mismatch repair	C7D0N7	EFBG_00070	DNA polymerase III delta prime subunit	5.222
translation process	Q831Q7	*rpsT*	30S ribosomal protein S20	—
translation process	Q836V1	*ileS*	Isoleucine--tRNA ligase	—
translation process	X5NXZ6	*rsmA*	Ribosomal RNA small subunit methyltransferase A	—
translation process	S4CQS9	D920_02813	Putative ribosomal protein S1	—
—	A0A3N3S475	EGW70_04250	Uncharacterized protein	0.339
—	X5NX31	P746_01209	Uncharacterized protein	0.316
—	V7ZPM0	T481_05220	Uncharacterized protein	0.240
—	X5NY46	P746_00971	Putative cytosolic protein	14.635
—	A0A059MZE5	HMPREF2097_02694	Uncharacterized protein	11.228
—	A0A059MZB7	HMPREF2097_02497	Uncharacterized protein	2.723
—	A0A059N0I6	HMPREF2097_01813	Uncharacterized protein	2.061
—	X5NWD9	P746_01417	Uncharacterized protein	—
—	A0A059MWP0	HMPREF2097_03580	Uncharacterized protein	—
—	X5NV03	P746_01160	Putative Membrane Spanning Protein	—

—	A0A0M2ADU4	UMC_00967	Uncharacterized protein	—
—	V7ZKT3	T481_16070	Cell surface protein	—
—	X5NYW4	P746_00292	Uncharacterized protein	—
—	X5NTY5	P746_01792	Putative membrane associated protein	—
—	C7CZ08	EFBG_02356	Uncharacterized protein	—
—	V7ZPB1	T481_07635	Uncharacterized protein	—
—	S4FJF5	D351_01067	Uncharacterized protein	—
—	A0A0M2A9P5	UMC_02204	Uncharacterized protein	—
—	A0A0M2AA44	UMC_02232	CAP_assoc_N domain-containing protein	—
—	S4FNQ9	D351_00708	Uncharacterized protein	—
—	A0A855P3A6	CUM81_13125	Uncharacterized protein	—
—	A0A1G1SA78	EU507_05790	Uncharacterized protein	—
—	A0A8D9IJU5	WE0254_00987	Uncharacterized protein	—
—	R3JMW3	WOK_00191	Uncharacterized protein	—
—	V7ZPY7	T481_09460	Uncharacterized protein	—
—	F6KLM3	pLG2-0069	Phage infection protein (Fragment)	—

Table S3 Common metabolites shared by MVs under pH 7.0 and pH 9.0 conditions

Metabolite class	ID	Metabolite name
Prenol lipids	M449T92	Geranylgeranyl pyrophosphate
Prenol lipids	M445T338	Menaquinone 4
Steroids and steroid derivatives	M465T25	Cholesteryl sulfate
Steroids and steroid derivatives	M359T237	Aldosterone
Organooxygen compounds	M421T500	α,α'-trehalose 6-phosphate
Organooxygen compounds	M397T301	Kasugamycin
Organooxygen compounds	M209T11	2,4-diacetylphloroglucinol
Organooxygen compounds	M540T428	Apramycin
Organooxygen compounds	M199T528	D-erythrose 4-phosphate
Organooxygen compounds	M734T182	Erythromycin
Organooxygen compounds	M528T449	Hygromycin b
Organooxygen compounds	M397T301	Kasugamycin
Organooxygen compounds	M336T292_2	Validamycin a
Organooxygen compounds	M219T196	D-glucuronate
Organooxygen compounds	M91T17	Glycerol
Organooxygen compounds	M668T482	Acarbose
Organooxygen compounds	M162T108	D-mannosamine
Organooxygen compounds	M87T70	Diacetyl
Organooxygen compounds	M204T589	N-acetyl-D-glucosamine
Organooxygen compounds	M129T110	Quinate
Organooxygen compounds	M117T354_1	Xylitol

Benzene and substituted derivatives	M138T241	4-aminobenzoate
Benzene and substituted derivatives	M181T73	4-hydroxyphenylpyruvate
Benzene and substituted derivatives	M103T85	Phenylacetaldehyde
Benzene and substituted derivatives	M91T34	Benzyl alcohol
Benzene and substituted derivatives	M121T228	Tyramine
Organonitrogen compounds	M104T301_2	Choline
Organonitrogen compounds	M114T50	Agmatine
Organonitrogen compounds	M162T4	L-carnitine
Organonitrogen compounds	M392T36	Guanidinopropionic acid
Organonitrogen compounds	M225T278	Porphobilinogen
Fatty Acyls	M340T58	Docosanamide
Fatty Acyls	M338T34_2	Erucamide
Fatty Acyls	M103T314	Isovaleric acid
Fatty Acyls	M103T449	Valeric acid
Fatty Acyls	M279T150	gamma-linolenic acid
Fatty Acyls	M89T36	Butanoic acid
Fatty Acyls	M233T36_2	Cis-10-heptadecenoic acid
Fatty Acyls	M257T27	Hexadecanoic acid
Fatty Acyls	M279T36	Linolenic acid
Fatty Acyls	M209T160	Myristoleic acid
Fatty Acyls	M297T49	Oleic acid methyl ester

Fatty Acyls	M256T64	Palmitamide
Fatty Acyls	M283T48_2	Octadecanoic acid
Fatty Acyls	M143T60_2	Octanoic acid
Fatty Acyls	M281T44	Oleic acid
Fatty Acyls	M255T50_2	Palmitic acid
Fatty Acyls	M241T45	Pentadecanoic acid
Fatty Acyls	M171T56	Capric acid
Fatty Acyls	M199T53	Dodecanoic acid
Carboxylic acids and derivatives	M118T302_2	Betaine
Carboxylic acids and derivatives	M561T341_2	Desferrioxamine
Carboxylic acids and derivatives	M296T179	3-oxo-c12 homoserine lactone
Carboxylic acids and derivatives	M184T269	3-oxo-c4-homoserine lactone
Carboxylic acids and derivatives	M116T24	Acetylglycine
Carboxylic acids and derivatives	M384T454	Actinonin
Carboxylic acids and derivatives	M397T252_2	Agaric acid
Carboxylic acids and derivatives	M289T167	Argininosuccinic acid
Carboxylic acids and derivatives	M132T447	Aspartic acid
Carboxylic acids and derivatives	M371T109	Biocytin

Carboxylic acids and derivatives	M145T337	DL-glutamine
Carboxylic acids and derivatives	M141T62	Ectoine
Carboxylic acids and derivatives	M146T414	Glutamic acid
Carboxylic acids and derivatives	M132T421	L-aspartic acid
Carboxylic acids and derivatives	M152T51	L-cysteinesulfinic acid
Carboxylic acids and derivatives	M130T258	L-Isoleucine
Carboxylic acids and derivatives	M148T58	L-methionine
Carboxylic acids and derivatives	M187T244	N-acetylglutamine
Carboxylic acids and derivatives	M131T147	Ornithine
Carboxylic acids and derivatives	M444T451	Tetrahydrofolate
Carboxylic acids and derivatives	M407T274	Lincomycin
Carboxylic acids and derivatives	M89T63	Isobutyric acid
Carboxylic acids and derivatives	M90T55	Sarcosine
Peptidomimetics	M556T439	Valinomycin
Peptidomimetics	M712T467_2	Microcolin c
Peptidomimetics	M473T26	Amastatin

Peptidomimetics	M962T436	Hoiamide b
Peptidomimetics	M697T138	Izenamide c
Peptidomimetics	M932T475	Microcystin la

Table S4 Significantly different metabolites of *E. faecalis* EVs at pH 9.0

Metabolite class	ID	Metabolite name	Fold change
Glycerophospholipids	M748T38	1-palmitoyl-2-oleoyl-phosphatidylglycerol	4.293
Glycerophospholipids	M757T167	1,2-dioleoyl-sn-glycero-3-phosphoethanolamine-n-methyl	0.183
Glycerophospholipids	M722T93	1,2-dipalmitoyl-sn-glycero-3-phospho-(1'-rac-glycerol)	4.836
Glycerophospholipids	M743T178	Pe 36:2	0.427
Glycerophospholipids	M693T98	Pg 30:0	5.733
Glycerophospholipids	M719T90	Pg 32:1	2.653
Glycerophospholipids	M926T182	Pi(18:0/7-hdohe)	2.468
Glycerophospholipids	M578T69	1-palmitoyl-2-oleoyl-sn-glycerol	3.685
Glycerophospholipids	M313T181	1-palmitoylglycerol	2.370
Glycerophospholipids	M552T88	1,2-dihexadecanoyl-sn-glycerol	3.728
Piperidines	M129T251	4-piperidinecarboxamide	2.679
Piperidines	M338T123	1-piperidinepropanol, .alpha.-(4-hydroxyphenyl)-.beta.-methyl-4-(phenylmethyl)-, (.alpha.r,.beta.s)-	0.173
Keto acids and derivatives	M181T367	2-Oxoadipic acid	1.428
Benzene and substituted derivatives	M135T236	3-methylbenzoate	2.701
Benzene and substituted derivatives	M110T696	2-aminophenol	1.985
Organooxygen compounds	M127T352	Glyceric acid	1.500

Organonitrogen compounds	M184T164	Phosphocholine	0.244
Pyrans	M141T354	Kojic acid	0.566
Triazines	M186T234	Metamitron-desamino	5.771
Sphingolipids	M618T36	N-palmitoyl-d-erythro-dihydroceramide-1-phosphate	0.384
Fatty Acyls	M157T59	Nonanoic acid	0.708
Fatty Acyls	M282T34_2	Oleamide	0.688
Fatty Acyls	M111T604	Suberic acid	1.896
Fatty Acyls	M283T34_2	Trans-vaccenic acid	0.692
Carboxylic acids and derivatives	M320T237_2	Phe-arg	0.212
Carboxylic acids and derivatives	M320T34_2	Linoleoylglycine	1.793
Carboxylic acids and derivatives	M437T35	Met-Met-Arg	1.713
Prenol lipids	M285T183	Isopimaric acid	2.357
Glycerophospholipids	M798T71	1,2-dioleoyl-sn-glycero-3-phospho-rac-1-glycerol	2.451
Lactones	M284T136	15-deoxy-goyazensolide	0.227
Indoles and derivatives	M148T664	3-methyloxyindole	1.775
Diazinanes	M202T293_1	4-(1-piperazinyl)-1h-indole	4.749
Phenols	M136T89	Dl-octopamine	0.217
Nucleosides, nucleotides, and analogues	M298T98	S-methyl-5'-thioadenosine	0.236
Quinolines and derivatives	M206T164	Xanthurenic acid	0.343

-	M592T72	2-lahpda [dmed-fahfa]	2.641
-	M523T89	2-mohma [dmed-fahfa]	7.274

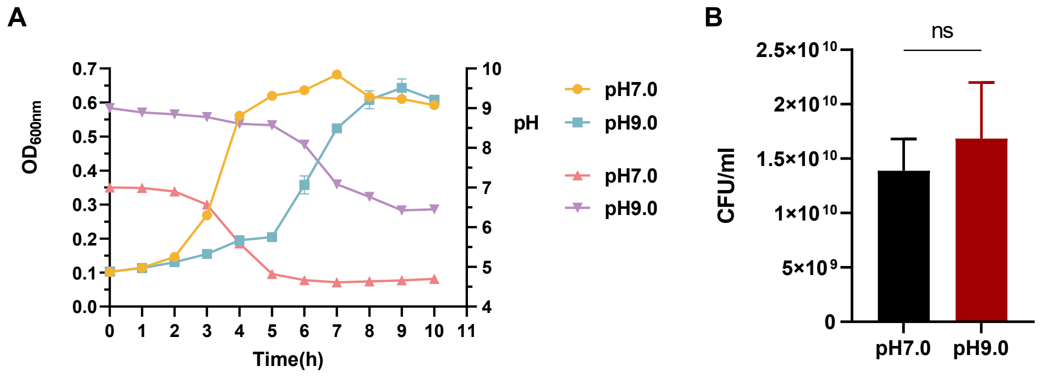

Figure S1 A) Growth curves and pH changes upon bacterial growth of *E. faecalis* under different pH conditions. B) *E. faecalis* colony counts after 9h of growth under different pH conditions. n = 3. The experiment was repeated three times; ns: non-significant.

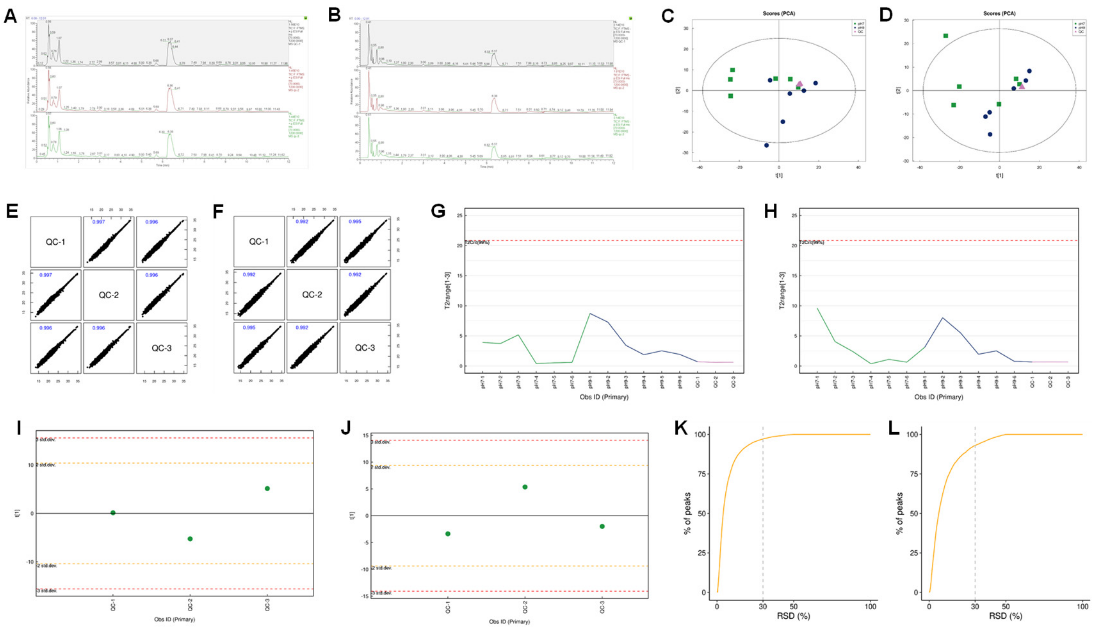

Figure S2 A-B) Total ion current chromatogram of QC samples, including positive ion mode (A) and negative ion mode (B). The abscissa represents the retention time of each chromatographic peak, while the ordinate represents the peak intensity value. The response intensities and retention times of each chromatographic peak basically overlap. C-D) Principal component analysis of overall samples. In the positive (C) and negative (D) ion modes, the samples within each group have a high degree of aggregation, and there is a distinct separation between groups. E-F) Correlation of QC samples. Pearson correlation analysis was performed on the QC samples in the positive (E) and negative

(F) ion modes. The correlation coefficient > 0.9 indicates a good correlation. G-H) Hotelling's T2 test of overall samples. Samples in the positive (G) and negative (H) ion modes were tested through multivariate variable modeling. The results show that all samples are within the 99% confidence interval. I-J) Multivariate control chart of QC samples. The results indicate that the fluctuations of QC samples in both the positive (I) and negative (J) ion modes are within ±3 standard deviations. K-L) Relative standard deviation (RSD) of QC samples. In the QC samples under the positive (K) and negative (L) ion modes, the proportion of the number of peaks with an RSD ≤ 30% to the total number of peaks in the QC samples is over 70%, indicating that the instrument analysis system has good stability.

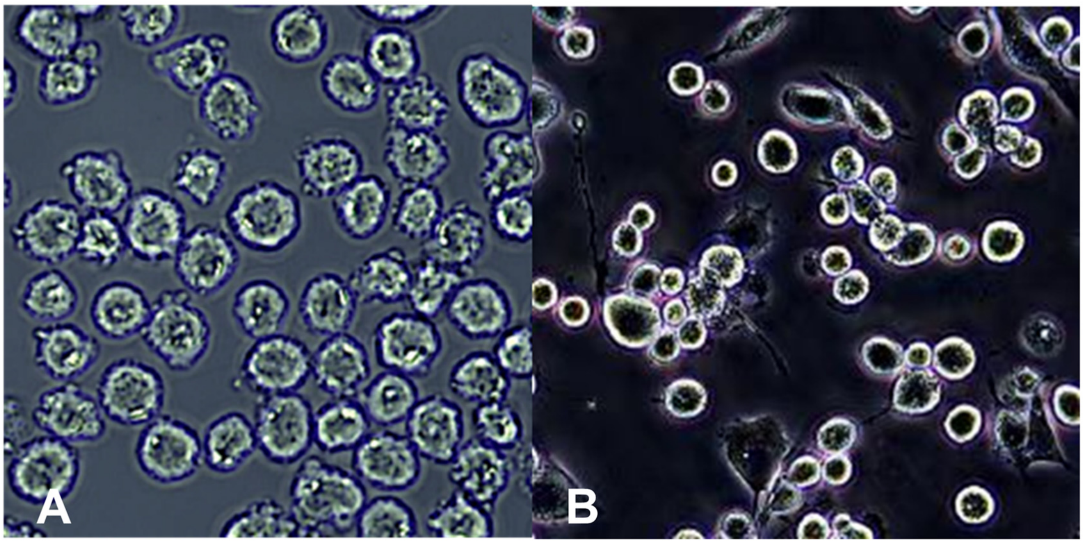

Figure S3 THP-1 cells were observed as rounded suspension cells (A) under light microscope. After PMA induced macrophages to differentiate, without treatment with *E. faecalis* MVs, dTHP-1 cells showed adherent growth, irregular shape, and visible pseudopods (B).

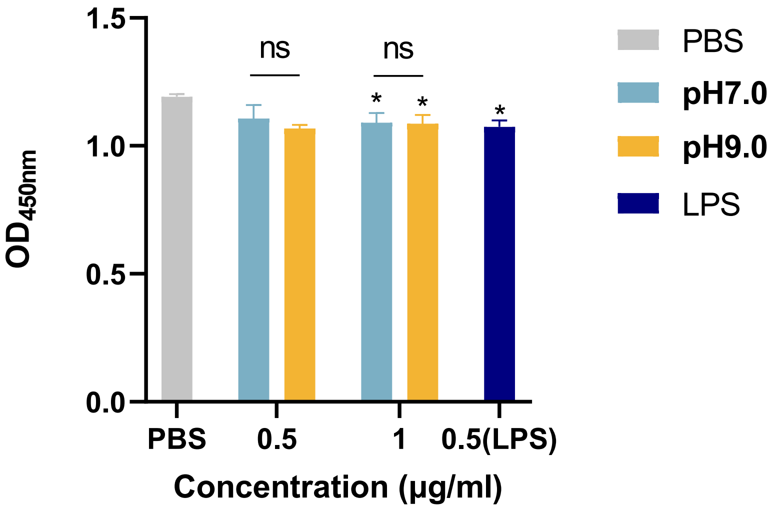

Figure S4 Effects of *E. faecalis* MVs on dTHP-1 cell viability under different pH conditions. n = 3. The experiment was repeated three times; *$p < 0.05$, ns: non-significant.
